# A novel homozygous exon2 deletion of *TRIM32* gene in a Chinese patient with sarcotubular myopathy: A case report and literature review

**DOI:** 10.17305/bjbms.2020.5288

**Published:** 2021-08

**Authors:** Xiao-Jing Wei, Jing Miao, Zhi-Xia Kang, Yan-Lu Gao, Zi-Yi Wang, Xue-Fan Yu

**Affiliations:** 1 Department of Neurology and Neuroscience Center, The First Affiliated Hospital of Jilin University, Jilin, China; 2 Department of Neurology, the Yan’an People’s Hospital, Yan’an, China; 3 Department of Neurology, The First Affiliated Hospital of Binzhou Medical University, Binzhou, China

**Keywords:** Sarcotubular myopathy, *TRIM32;* vacuoles, homozygous deletion, case report

## Abstract

Sarcotubular myopathy (STM) is a rare autosomal recessive myopathy caused by *TRIM32* gene mutations. It is predominantly characterized by the weakness of the proximal limb and mild to moderate elevation of creatine kinase levels. In this study, we describe a 50-year-old Chinese man who exhibited a proximal-to-distal weakness in the muscles of the lower limbs and who had difficulty standing up from a squat position. The symptoms gradually became more severe. He denied a history of cognitive or cardiological problems. The patient’s parents and children were healthy. Histopathological examination revealed dystrophic changes and irregular slit-shaped vacuoles containing amorphous materials. Whole-exome sequencing consisting of protein-encoding regions of 19,396 genes was performed, the results of which identified one novel homozygous 2kb deletion chr9.hg19: g.119460021_119461983del (exon2) in the *TRIM32* gene. This was confirmed at the homozygous state with quantitative real-time polymerase chain reaction. Here, we present a Chinese case of STM with one novel mutation in *TRIM32* and provide a brief summary of all known pathogenic mutations in *TRIM32*.

## INTRODUCTION

Sarcotubular myopathy (STM; MIM 268950) is a rare autosomal recessive disease characterized predominantly by weakness of the proximal limb. Mutations in the *TRIM32* gene, which encodes a protein consisting of 653 amino acids residues, are often associated with STM and the limb-girdle muscular dystrophy R8 (LGMDR8, previously known as LGMD2H; MIM 254110) [1], two conditions that present as different severities of the same disease [2]; Bardet Biedl syndrome presents a different phenotype due to one variant (P130S) in the B-box region, independently from muscular phenotypes [3]. Since the first case was reported in Jerusalem in 1973, STM has only been described in 28 patients across the world [2,4-10].

*TRIM32* is a member of the tripartite motif (TRIM) family and is composed two exons, with the entire open reading frame located within exon2. The TRIM32 protein sequence contains several conserved N-terminal motifs including a RING-finger, a B-box, and a coiled-coil region, followed by six NHL repeats at the C-terminus [11]. Various mutations have been reported to cause *TRIM32*-related myopathy, and the majority of these patients are of Hutterite origin and overwhelmingly homozygous for the c.1459G >A (p. D487N) mutation [1,12]. However, additional mutations that cause LGMDR8/STM have since been documented in different populations [6,8-10,13-15]. To date, no disease-causing mutations in the *TRIM32* gene have been reported in the Asian population. A total of 27 different mutations have been reported in patients with *TRIM32*-related myopathy, including one nonsense, ten missense, nine frameshifts, two small deletion, and five large deletion variants (Table S1). Apart from the most frequent D487N mutation and all the recently identified single nucleotide pathogenic variants in the C-terminal NHL domain, a relatively high frequency of frameshift mutations have been reported outside the NHL repeats.

Herein, we identified a Chinese patient with a new homozygous deletion mutation in exon2 and described the clinical, pathological, and image-based findings of his clinical assessment. This is the first independent report of *TRIM32*-mutated STM in China.

## CASE REPORT

A 50-year-old male was admitted to our hospital complaining of slowly progressing weakness. He stated that when he was 4 years old, his mother noticed that he was not to be able to run as fast as his peers. He was the first of three children of his non-consanguineous parents, and both his siblings and his parents were healthy. No other muscular symptoms were noted throughout his childhood. In the 2 years leading up to his evaluation, he noticed mild weakness in his legs and difficulty getting up from a squatting position. These symptoms gradually became more severe, until he presented at age 50 with proximal-to-distal weakness in the muscles of the lower limbs. He has never experienced cognitive or cardiologic problems.

**FIGURE S1:**
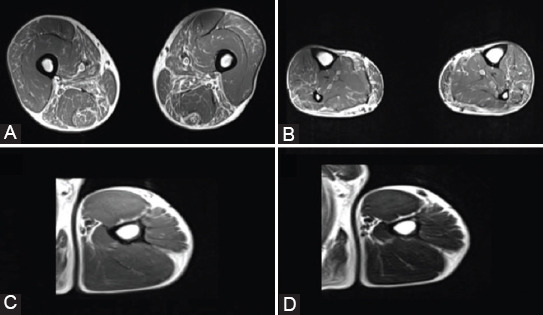
Magnetic resonance imaging (MRI). Axial T1-weighted MRI scans are in the lower limb, and axial T1-weighted and T2-weighted MRI scans are in the upper limb. A more focal pattern of fatty degeneration was observed, mainly involving the biceps femoris and semimembranosus muscle in the thigh, while the gracilis, sartorius, and quadriceps muscles were spared (A); severe fatty replacement of the gastrocnemius was observed in the patient (B). In the upper limbs, no significant changes are visible yet (C-D).

Neurological examination revealed mild proximal muscle weakness in the lower extremities and the toe extensor muscles (MRC grade 4). The Gower’s sign test was positive and his tendon reflexes remained present but weak. There was no evidence of calf hypertrophy, scapular winging, or facial, and ocular impairment. Laboratory testing revealed slightly elevated creatine kinase levels (2× normal). Electromyography abnormalities of myopathic changes were found in both the vastus medialis and tibialis muscles. Echocardiograph and electrocardiogram results were normal. A magnetic resonance imaging (MRI) of the patient’s muscles revealed a preferential fatty infiltration in the posterior compartment of the patient’s thigh, with marked sparing in the gracilis, sartorius, and quadriceps muscles (Figure S1A). There was more focal involvement in the gastrocnemius muscles, and the remaining muscle groups were mildly involved (Figure S1B). The upper limb muscles demonstrate minimal fatty infiltration and no distinct involvement pattern (Figure S1C-D).

**FIGURE 1:**
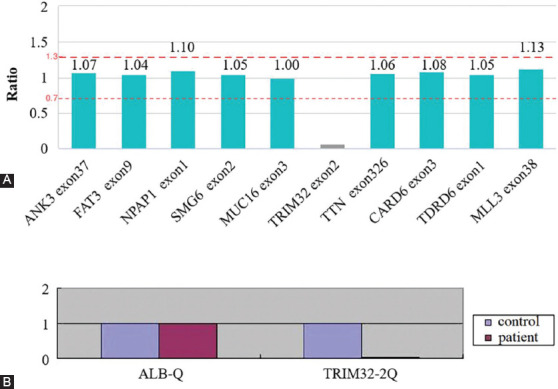
*TRIM32* deletion mapping and mRNA expression. Whole exome sequence data showing the homozygous deletion of the coding region of exon 2 in the *TRIM32* gene, which leads to a total absence of amplification and a ratio of zero for the probe covering this region (A). Quantitative real-time polymerase chain reaction (qRT-PCR) analysis of *TRIM32* messenger RNA expression in blood. Messenger RNA levels were quantified with real-time PCR and normalized to the levels of ALB. A significant reduction in real-time PCR quantification of *TRIM32* gene expression is observed in the patient’s blood compared with unaffected controls (B).

Peripheral blood samples were obtained with informed consent from three members of the family. Genomic DNA was isolated from these samples according to standard techniques. All known protein-encoding regions, including a total of 19,396 genes, were captured and enriched. The enriched libraries were sequenced on an Illumina NovaSeq 6000 (Illumina, USA), which generated data covering 99.9% of the sequence at 300×. The sequencing reads were aligned on a reference human genome (hg19) using Burrows-Wheeler Aligner software. The Genome Analysis Toolkit (GATK) was used to detect single-nucleotide variants and indels of the reads. Whole exome sequencing was performed at Chigene Medical Research Centre (Beijing, China). It turned out that the patient had homozygous deletion of a 2kb region, chr9.hg19: g.119460021_119461983del, in exon 2 of the *TRIM32* gene (Figure 1A). The presence of this homozygous deletion was confirmed with quantitative real-time polymerase chain reaction (Figure 1B). The patient’s parents were heterozygous for the mutation. This novel mutation was neither observed in 200 healthy Chinese controls, nor four public databases, including dbVar, ClinVar, the Database of Genomic Variants (http://dgv.tcag.ca/dgv/app/home), and the Leiden Open Variation Database (www.lovd.nl/TRIM32).

**FIGURE 2:**
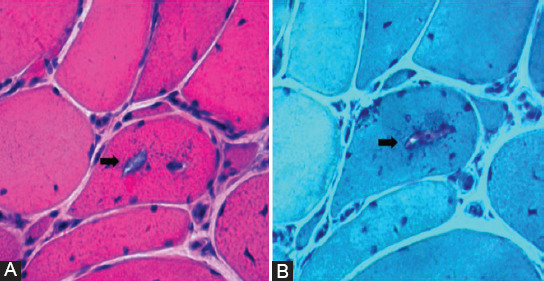
Histopathologic features. Hematoxylin and eosin (H&E) staining on the patient’s muscle biopsy showed an increased number of internal nuclei, fiber size variations, and vacuoles (arrow) in scattered muscle fibers (A); Gomori trichrome staining displayed an increase in the density of basophilic material (arrow) in vacuoles (B).

After written informed consent was obtained from the patient, a gastrocnemius biopsy was conducted. Muscle biopsy revealed marked variation of the muscle fiber diameters, internalized nuclei, as well as necrotic and atrophic myofibers. Small and irregularly slit-shaped vacuoles containing basophilic material were observed throughout the sarcoplasm in scattered myofibers. The connective tissue of the muscle was moderately increased. However, no inflammatory cell infiltration was observed (Figure 2). Immunohistochemical staining showed that anti-dystrophin-N, -C, -R proteins, anti-α-, β-, γ-, δ-sarcoglycan proteins, calpain-3, and dysferlin levels were normal.

## DISCUSSION

Our patient’s STM diagnosis is based on limb-girdle weakness, the presence of vacuoles in his skeletal muscle biopsy, and genetic testing. To our the best of knowledge, this is the first report outlining the diagnostic characteristics of STM in a patient of Chinese ethnicity, and the 29^th^ report worldwide that assesses STM.

**FIGURE 3:**
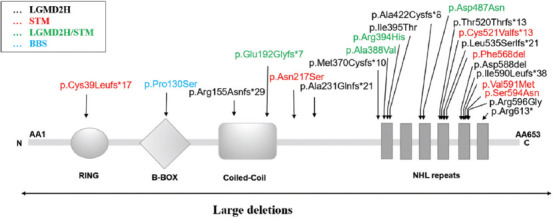
Location of all detected variants associated with *TRIM32*-related myopathy patients. The arrows indicate the position of mutations identified in the *TRIM32* protein structure. Mutations associated with LGMD2H and STM are shown in black and red, respectively; mutations that may show up in the LGMD2H and STM phenotypes are indicated in green; whereas mutation corresponding to Bardet–Biedl syndrome is indicated in blue.

The number of reports, including systematic literature reviews, on pathogenic *TRIM32* mutations is limited. Since *TRIM32* mutation-related disorders were first described by Frosk et al. in 2002, 81 total cases have been formally reported. *TRIM32* gene mutations and their associated phenotypes are summarized in Figure 3 and Table S1. *TRIM32* mutations are most commonly associated with LGMDR8 (68% [55/81] of the cases), followed by STM (30% [24/81] of the cases). Furthermore, STM generally has an earlier onset characterized by more severe weakness than LGMDR8 [12,15,16]. As previously reported by Nectoux et al., although the proband became wheelchair-bound in the late course of his disease, a relatively mild phenotype, as in the current patient, has also been noted [17]. Our patient was still able to walk without support, suggesting that the clinical manifestation of individuals with homozygous deletions of *TRIM32* may not correlate with a higher severity in phenotype. Therefore, the relationship between the type of mutation and the resulting phenotype should be investigated further.

Nectoux et al. reported a patient with early-onset LGMDR8 and mild cognitive deterioration who had a homozygous 336 Kb deletion that encompassed the entire *TRIM32* gene and part of the *ASTN2* gene [17]. They postulated that the cognitive disability could be related to a complete loss of function of *TRIM32/ASTN2* activity. Conversely, our patient did not have any evidence of cognitive impairment, including no abnormal difficulty with concentration or weakness in immediate memory for his age. This discrepancy in phenotypes suggests that cognitive impairment should be associated with *ASTN2* loss of function rather than *TRIM32* loss of function. However, further investigation is needed to verify this novel finding. Together with the previously published report, our results indicate that a homozygous deletion variant is associated with pathogenicity of *TRIM32*-related myopathy.

In summary, we report a novel *TRIM32* mutation in a Chinese patient with STM and describe the differences in clinical phenotype between a homozygous deletion mutation and other previously identified mutations. This observation suggests that *TRIM32* inactivation may not influence cognitive impairment in STM patients. We also provided MRI evidence of the upper limb muscles from a TRIM32 mutated patient. This finding broadens the mutational and phenotypic spectra of *TRIM32*-related myopathies and can guide proper genetic counseling for pre- and post-natal screenings in the future.

## Figures and Tables

**TABLE S1:**
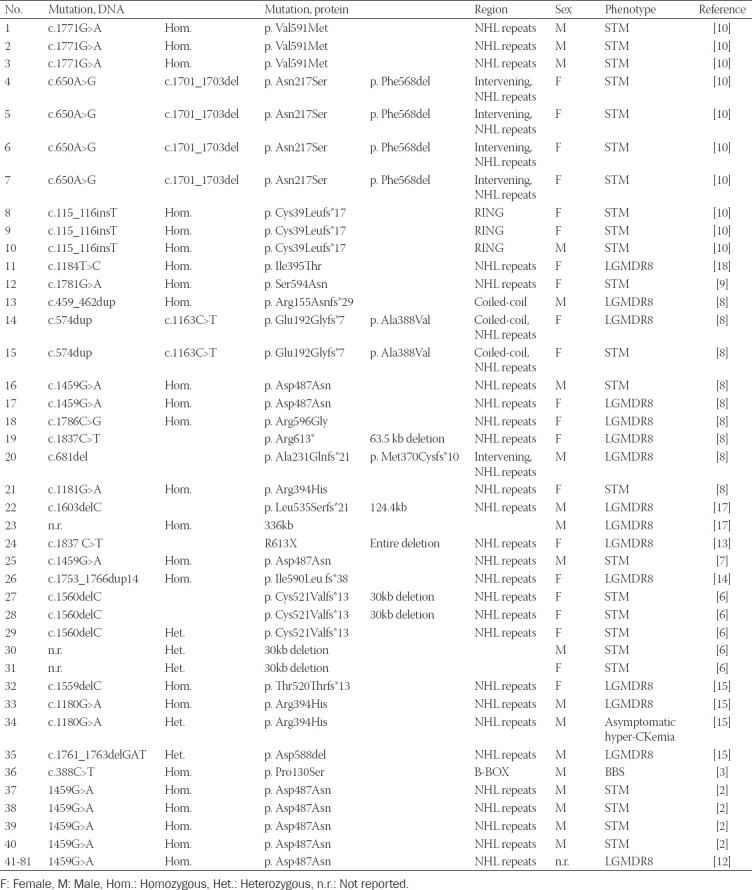
Summary of 81 cases of TRIM32 gene mutation in the literature
